# Preserving RNA integrity and morphology in whole rat allograft hindlimb sections: an optimized protocol for spatial transcriptomics and immunofluorescence

**DOI:** 10.3389/fmolb.2026.1910122

**Published:** 2026-10-19

**Authors:** Marie Dreck, Elena Grabis, Laura Schneider, Maike Lind, Philipp Heinrichs, Thierry Christmann, Nicolas Neidert, Teresa Z. Brose, Sheena Kreuzaler, Franziska Pankratz, Dieter H. Heiland, Steffen U. Eisenhardt, Julia Thomé

**Affiliations:** 1 Department of Plastic and Hand Surgery, Medical Center and Faculty of Medicine, University of Freiburg, Freiburg, Germany; 2 Department of Neurosurgery, Medical Center and Faculty of Medicine, University of Freiburg, Freiburg, Germany; 3 Translational Neurosurgery, Friedrich-Alexander University Erlangen Nuremberg, Erlangen, Germany

**Keywords:** immunofluorescence staining, rat hindlimb, RNA integrity, spatial transcriptomics, vascularized composite allograft

## Abstract

Vascularized composite allotransplantation holds great promise in reconstructive surgery, offering the potential to significantly improve the quality of life for patients with extensive facial injuries or limb amputations. However, this type of transplantation remains challenging due to the high complexity of multiple tissue types and the diverse immunological responses within the graft. For investigation of rejection patterns valid experimental models, including rodent hindlimb transplantation, are crucial. Spatial transcriptomics provides a powerful approach to investigate gene expression patterns associated with ongoing rejection in their spatial context. To date, spatial transcriptomics has not yet been described for rat hindlimb transplantation. In this study, we describe an optimized protocol for spatial transcriptomics and immunofluorescence staining of rat hindlimb cross-sections. The rat model enables a detailed investigation of the spatial cellular organization across the entire transplanted limb, encompassing muscle, skin, nerve, and vascular structures. We present a refined workflow encompassing tissue embedding, cryosectioning, immunofluorescence staining, permeabilization timing, and RNA quality control, followed by the workflow of the spatial transcriptomics procedure. This protocol establishes a reproducible and robust framework for the spatial characterization of vascularized composite allografts and provides a foundation for future spatial studies of immune-mediated rejection and musculoskeletal disease in rodent limb models.

## 1 Introduction

Vascularized composite allografts (VCAs) are transplants of functional anatomical units composed of multiple distinct tissue types, including skin, muscle, nerves, bone, tendons, adipose tissue and blood vessels, which are transferred as integrated tissue compartments (Pomahac et al., 2012).

Clinically, VCAs represent a therapeutic option in complex reconstructive surgery. Prominent examples include upper- and lower- extremity transplantation, as well as facial transplantation (Devauchelle et al., 2006; Dubernard et al., 1999; Fattah et al., 2011). Additional applications include abdominal wall, laryngeal and reproductive organ transplantation (Brännström, 2018; Levi et al., 2003; Strome et al., 2001).

Unlike solid organ transplantation, VCAs are rarely life-saving procedures but rather aim to restore form and function after severe soft tissue or musculoskeletal injuries, amputations or extensive tissue loss (Kiwanuka et al., 2013; Morris et al., 2007). However, the unique compositional complexity of VCAs renders them particularly vulnerable to immune-mediated rejection. The presence of highly immunogenic skin, combined with multiple additional tissue compartments, creates a heterogeneous and immunologically active graft environment (Ashraf et al., 2024; Madariaga et al., 2015). Consequently, VCAs are immunologically more challenging than most solid organs and still lack complete understanding of the underlying immunopathology. Monocytes are increasingly recognized as key regulators of transplant immunity due to their functional heterogeneity and their ability to bridge innate and adaptive immune responses (Cros et al., 2010; Thomé et al., 2025a; Thomé et al., 2025b; Wong et al., 2011). To enable spatial tracking and transcriptional characterization of recipient-derived monocytes within the composite graft, we therefore employed hCD68-GFP transgenic recipient rats in our hindlimb VCA model.

Despite the existence of clinical diagnostic criteria, a comprehensive spatial and transcriptional understanding of immune cell dynamics within the composite graft during rejection is still lacking. Spatial transcriptomics offers a powerful approach to address this knowledge gap by enabling the spatially resolved analysis of gene expression across entire tissue sections, thereby linking immune cell localization with transcriptional activity within the complex VCA microenvironment.

Multiple VCA models have been established across different species, including pig forelimb transplantation (Fries et al., 2016), mouse hindlimb composite allotransplantation (Sucher et al., 2010), canine composite facial and scalp transplantation (Shengwu et al., 2007), primate fibula VCA (Mundinger et al., 2011) and rat hindlimb transplantation (Doi, 1979; Fleissig et al., 2019). Among these, the rat hindlimb model is particularly well characterized and widely used for studying rejection biology (Dzhonova et al., 2018; Hautz et al., 2013; Olariu et al., 2017; Sutter et al., 2019; Unadkat et al., 2017). These animal models provide important insights into immune responses and immunomodulatory strategies, yet most analyses have focused on either macroscopic graft changes or conventional histology, offering limited resolution of the complex cellular interactions occurring within the composite tissue.

In parallel, spatial transcriptomics has emerged as a powerful technology enabling spatially resolved gene expression profiling and has been successfully applied to various tissues, including tumors (Fang et al., 2023), lung (Jiang et al., 2024), ureter (Fink et al., 2022) and skin (Foster et al., 2021). Despite these advances, the application of spatial transcriptomics to large, heterogeneous composite tissues such as vascularized allografts remains limited. To date, no spatial transcriptomic analysis has been established for whole-limb VCA models.

A major obstacle to implementing spatial transcriptomics in whole-limb VCA models lies not only in the analytical platform itself, but in the complex pre-analytical processing of large, heterogeneous tissue. The rat hindlimb comprises structurally and mechanically distinct compartments that require careful coordination of tissue harvest, ischemia control, cryopreservation, embedding, sectioning, and permeabilization. Inadequate handling at any of these stages may compromise RNA integrity, section morphology or spatial resolution. Furthermore, differences in tissue density and composition complicate uniform permeabilization and RNA release across compartments. Standard spatial transcriptomic workflows, which were developed for smaller and more homogeneous tissue samples, are therefore not readily transferable to intact composite grafts. Moreover, commonly used protocols do not reliably preserve endogenous fluorescent reporter signals, limiting the integration of spatial transcriptomics with immune cell tracking approaches.

To overcome these challenges, we developed and refined a dedicated tissue-processing workflow for rat hindlimb VCA model using hCD68-GFP transgenic recipients, enabling direct visualization of host-derived macrophages within the graft (Diaz et al., 2016). This study focuses on establishing a reproducible protocol encompassing tissue harvest, controlled cryopreservation, bone removal, cryosectioning, permeabilization optimization, RNA quality control, and preservation of endogenous GFP signals. Rather than emphasizing downstream biological interpretation, the primary objective of this work is to provide a robust methodological framework that enables reliable spatial transcriptomic and immunofluorescence analyses in large, heterogeneous composite grafts.

## 2 Materials and methods

### 2.1 Surgical procedures and experimental model

Tissue samples were obtained from replanted and transplanted rat hindlimbs (n = 8). Lewis rats were used as hindlimb donors, while transgenic Wistar Kyoto hCD68-GFP rats served as recipients, allowing the identification and spatial tracking of host-derived macrophages within the transplanted tissue (Diaz et al., 2016; Turner-Stokes et al., 2020). All animals were male, 7–11 weeks old and weighing 126–250 g at the time of surgery. Surgery was performed under general anesthesia with 2 vol% isoflurane and buprenorphine (0.05 mg/kg, subcutaneously) and additional regional anesthesia with 2 mg/kg ropivacaine to prevent neuropathic pain. Tissue harvest occurred between day 18 and 90 after surgery. Figure 1 shows an overview of the experimental design.

**FIGURE 1:**
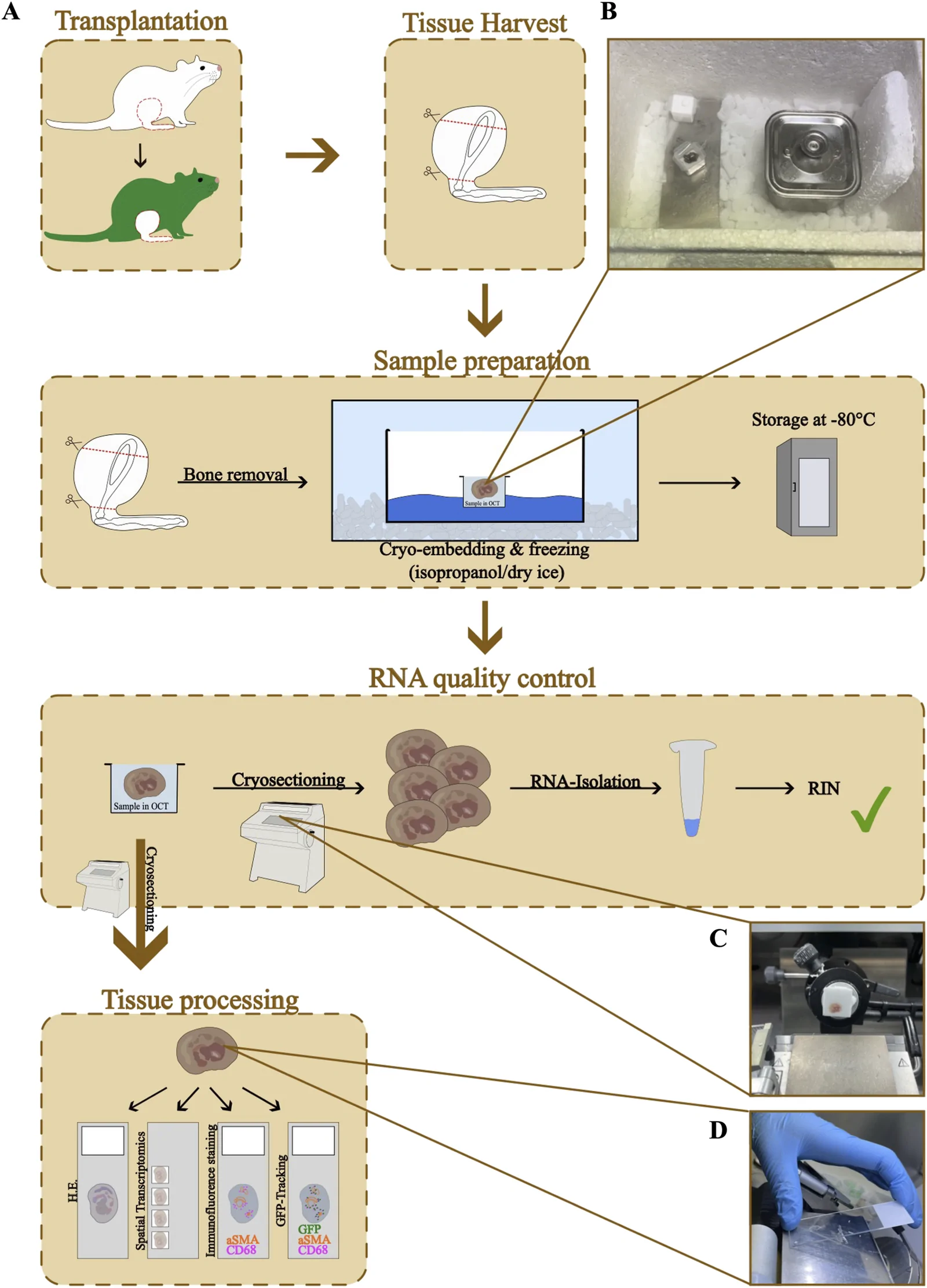
Schematic overview of the optimized workflow for spatial transcriptomics and immunofluorescence analysis of rat hindlimb VCA tissue. **(A)** The Illustration shows the key steps of the tissue processing pipeline, including tissue harvest, cryo-embedding and controlled freezing in an isopropanol bath on dry ice as the preferred freezing method, cryosectioning, RNA quality control and tissue processing. **(B)** Cryo-embedding and freezing setup using an isopropanol bath on dry ice **(C)** Sample mounted in the cryostat prior to cryosectioning. **(D)** Placement of the cryosection onto the microscope slide.

All animals were housed under standard conditions in the animal facility of the medical center of the University of Freiburg. All experiments were conducted according to the ethical policies and procedures and were approved by the ethics committee at the University of Freiburg, Germany (Nos. G-21/102 and G-24/009).

### 2.2 Sample acquisition and preparation

The rat hindlimb was shaved and treated with depilatory cream to remove any remaining hair, as residual hair can interfere with both the freezing process and subsequent microscopy. Subsequently, animals were euthanized under high-dose anesthesia (100 mg/kg Ketamin, 20 mg/kg Xylazin) via exsanguation in compliance with the German Animal Welfare Act. Ischemia tome was kept as short as possible to preserve RNA integrity. Following perfusion with heparin solution (100 IU/mL), the limb was dissected distal to the tibial plateau using scissors and forceps. An additional transection proximal to the ankle joint was performed to isolate the lower leg (Figure 1). The tibia and fibula were then carefully removed by periosteal elevation, ensuring that the overall anatomical integrity of the hindlimb remained intact. Preliminary cryosectioning trials had shown that the presence of bone caused mechanical disruption of adjacent soft tissue, resulting in fragmented and uneven sections that were unsuitable for histological and spatial transcriptomic analysis. Consequently, all subsequent experiments were performed using bone-free hindlimb specimens to ensure consistent section quality and downstream data integrity.

### 2.3 Embedding and freezing

Cryo-embedding was initially evaluated using two freezing approaches: rapid freezing in liquid nitrogen and controlled freezing in an isopropanol bath on dry ice. Liquid nitrogen freezing resulted in reduced RNA integrity; therefore, controlled freezing in an isopropanol bath on dry ice was selected for all subsequent experiments. The cryo-embedding setup consisted of a pre-cooled isopropanol bath in a metal beaker placed in a Styrofoam box filled with dry ice. A cryomold was layered with OCT Tissue-Tek and partially frozen in the bath. The dissected hindlimb was positioned in the cryomold with the proximal side facing downward, while the distal end was stabilized with tweezers during initial freezing to maintain its orientation. Additional OCT medium was gradually added around the tissue until the specimen was fully embedded, while minimizing air bubble formation to facilitate smooth and intact cryosectioning. The sample was kept in the isopropanol bath until completely frozen and was then transferred to −80 °C for long-term storage.

### 2.4 Cryosectioning

The cryo-fixed rat hindlimb specimens were mounted in the cryostat and allowed to equilibrate for 30 min at −20 °C before sectioning at a thickness of 10 µm. Sectioning was performed starting from the proximal end of the sample. The surrounding OCT was then carefully reduced so that only a minimal margin remained around the specimen (Figure 1B). Final sections were collected using fine brushes to orient the samples and to remove any residual OCT from the edges. Microscope slides were pre-cooled to −20 °C to minimize the formation of freezing artifacts. The prepared tissue sections were then transferred onto either a Visium Spatial Gene Expression Slide or a Visium Spatial Tissue Optimization Slide. To ensure proper adhesion, the area of the slide corresponding to the placement site was gently warmed from the reverse side with a fingertip immediately before positioning the section. Precise alignment of the tissue within the 6.5 × 6.5 mm capture area was essential. Most cross-sections of the samples, especially distal to the knee joint, fit entirely within the capture area. Sections that exceeded the boundaries were carefully trimmed to ensure that all major tissue components were represented within the capture region. Consecutive sections were transferred in the same manner onto SuperFrost Plus slides for subsequent immunofluorescence staining.

### 2.5 RNA quality control

Total RNA was extracted from five 10 µm cryosections of rat hindlimb tissue to assess RNA integrity and overall sample quality. Two extraction protocols were used: the conventional phenol–guanidine thiocyanate–chloroform method and the Qiagen RNeasy Mini Kit (Qiagen, Hilden, Germany, Cat. No. 74106). For both protocols, tissue sections were homogenized in 0.5 mL TRIzol reagent, followed by chloroform-induced phase separation. For the conventional extraction, the aqueous phase was precipitated with isopropanol, washed with 75% ethanol, pelleted, DNase-treated, and finally resuspended in nuclease-free water. For the Qiagen RNeasy Mini Kit extraction, the aqueous phase was mixed with an equal volume of 70% ethanol and processed according to the manufacturer’s instructions, including RW1 and RPE washes and elution in 30 µL RNase-free water. The isolated RNA was stored at −80 °C, and RNA integrity was assessed using an Agilent TapeStation (Agilent Technologies, Santa Clara, United States).

### 2.6 Immunofluorescence staining

Consecutive 10 µm cryosections were used for immunofluorescence staining. To preserve the endogenous GFP signal from hCD68-GFP rats, fixation was performed according to Scandella et al. (2020), with modifications. Sections were kept moist at all times and immediately fixed in pre-warmed (37 °C) 4% paraformaldehyde (PFA, pH 7.4) for 15 min, followed by two 10 min PBS washes. Samples were then blocked and permeabilized in 5% goat serum with 0.1% Tween-20 in PBS for 1 h at room temperature.

Primary staining was performed sequentially with anti-GFP (1:100, FM264G, Alexa Fluor 488, Bio Legend, San Diego, CA, United States, AB_2563287) and anti-CD68 (1:100, ED-1, Unconjugated, Merck, Darmstadt, Germany, AB_177576) antibodies, each incubated overnight at 4 °C in a humidified chamber (100 µL per slide, sealed with Parafilm). After each incubation, slides were washed three times for 5 min in PBS. The secondary antibody, goat anti-mouse IgG (1:100, Polyclonal, Cy5, Thermo Fisher Scientific, Waltham, MA, United States, AB_10562712) was applied for 2 h at room temperature, followed by incubation with anti-αSMA (1:200, 1A4, Cy3, Merck, Darmstadt, Germany, AB_476856) for 6 h. Finally, sections were washed in PBS and mounted with VECTASHIELD Vibrance® Antifade Mounting Medium with DAPI under 60 mm coverslips. All samples were digitalized using the Axio Scan 7 slide scanner (Zeiss, Oberkochen, Germany) or imaged with an LSM980 MP AiryScan 2 confocal microscope (Zeiss, Oberkochen, Germany) under 10x or ×63 (oil) magnification. Confocal images were acquired with the pinhole set to 1 AU for all channels except GFP, which was acquired with a pinhole of 0.83 AU to improve optical sectioning and signal specificity.

### 2.7 Spatial transcriptomics workflow

We performed tissue optimization according to the Visium Spatial Gene Expression Reagent Kits—Tissue Optimization User Guide (CG000238 Rev E; 10x Genomics). Seven tissue sections were placed within the capture areas of the Visium Tissue Optimization Slide and stained with H&E. A 2 µL RNA droplet served as a positive control. Permeabilization enzyme was added sequentially to each tissue section to generate increasing incubation times, while one section remained untreated as a negative control. Following permeabilization, the slides were washed and incubated with reverse transcription master mix containing fluorescently labeled nucleotides, according to the manufacturer’s protocol. Tissue was subsequently enzymatically removed, leaving fluorescent cDNA bound to the slide oligonucleotides. Fluorescence microscopy images were compared with the corresponding H&E-stained sections to determine the optimal permeabilization time based on signal intensity and minimal diffusion. All fluorescence images used for the comparison of permeabilization times were acquired using an Axio Scan 7 slide scanner (Zeiss, Germany) equipped with a Plan-Apochromat 10×/0.45 objective. Identical acquisition settings were used for all samples, including the AF555 fluorescence channel, an exposure time of 35 ms, and a lamp intensity of 5%, to ensure comparability of fluorescence intensities across permeabilization conditions. Afterwards, fluorescence-positive area was assessed semiquantitatively in ImageJ by applying an identical intensity threshold and calculating the percentage of fluorescence-positive pixels relative to the total image area for each permeabilization condition. An incubation time of 33 min was identified as optimal.

The spatial gene expression experiment was carried out following the Visium Spatial Gene Expression Reagent Kits User Guide (CG000239 Rev G; 10x Genomics). Based on prior tissue optimization, the permeabilization time was set to 33 min (Figure 2). During permeabilization, released mRNA molecules hybridized to poly (dT) capture oligonucleotides on the Visium slide. Reverse transcription was subsequently performed using RT master mix for 45 min at 53 °C to generate spatially barcoded cDNA. Following tissue removal, second-strand synthesis and denaturation were performed according to the manufacturer’s instructions. cDNA amplification cycles were determined by qPCR based on the cycle threshold corresponding to approximately 25% of maximal fluorescence intensity. Amplified cDNA was purified using SPRIselect reagent and quantified using the Agilent TapeStation system. Library construction included enzymatic fragmentation, double-sided size selection, adapter ligation, sample index PCR, and final library purification following the standard Visium workflow. Final library fragment size was assessed using the Agilent TapeStation. Libraries were sequenced on Illumina NextSeq 1000/2000 and NovaSeq X Plus (PE150-10B) platforms.

**FIGURE 2:**
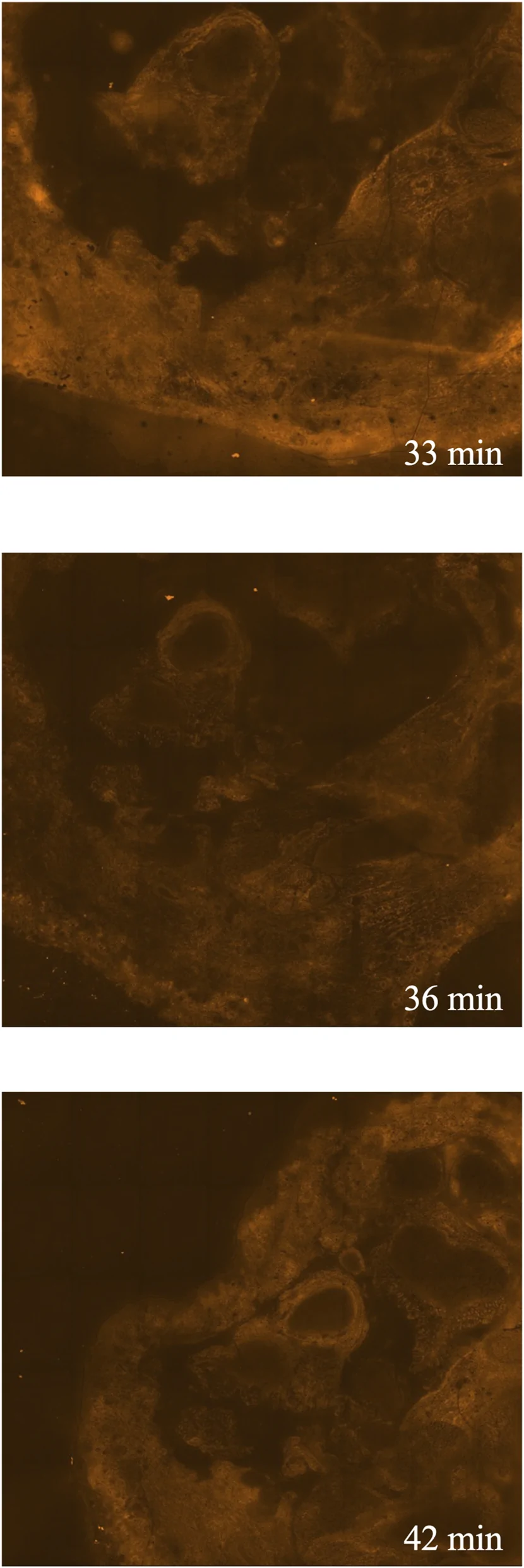
Optimization of tissue permeabilization for spatial transcriptomics. Representative immunofluorescence images of muscle tissue regions within rat hindlimb-cross sections illustrating the resulting TRITC signal intensities. All images were acquired using an Axio Scan 7 slide scanner (Zeiss, Germany) with identical microscope and acquisition settings. The strongest and most homogeneous fluorescence signal was obtained after 33 min of permeabilization.

### 2.8 Data processing

Raw sequencing reads were processed with Space Ranger (10x Genomics, Pleasanton, CA United States) to obtain spatially resolved gene expression matrices. Subsequent data analysis was performed using Seurat (Hao et al., 2024) and SPATA2 (Kueckelhaus et al., 2024), including visualization of spatial gene expression per spot and unsupervised clustering to identify transcriptionally distinct tissue regions.

For unsupervised clustering, 2,000 highly variable genes were identified, followed by principal component analysis. A nearest-neighbor graph was constructed using the first 20 principal components, followed by Louvain clustering at a resolution of 0.3. Differential expression analysis between spatial clusters was performed using the Wilcoxon rank-sum test. Differentially expressed genes were filtered using an adjusted p-value < 0.05, an average log2 fold change > 0.5, and expression in at least 10% of spots within the respective cluster. Representative marker genes were selected from the filtered genes based on differential expression and biological coherence to characterize the transcriptional identity of the spatial clusters.

Cell-type composition was inferred by integrating the spatial transcriptomics data with a published *Rattus norvegicus* single-cell transcriptomic dataset (Ma et al., 2020) using cell2location (Kleshchevnikov et al., 2022) and CytoSPACE (Vahid et al., 2023).

Endothelial cell enrichment was visualized spatially, and vascular structures were manually annotated on the corresponding H&E images. Vessel distances were calculated using the annotation screening functionality in SPATA2.

## 3 Results

### 3.1 Column-based RNA isolation improves RNA quality

RNA integrity was assessed using the RNA Integrity Number (RIN). RNA was isolated either with the conventional phenol–guanidine thiocyanate–chloroform protocol or with the Qiagen RNeasy Mini Kit. Samples processed with the conventional method yielded RIN values between 3.7 and 4.2 (Samples 1, 2, 3, 4), while those isolated with the Qiagen kit achieved higher RIN values between 6.0 and 6.9 (Samples 5, 6, 7, 8). Although none of the samples reached the RIN threshold of 7 recommended by 10x Genomics, subsequent spatial transcriptomic quality control demonstrated robust assay performance and high spatial signal integrity. Notably, these findings indicate that spatial transcriptomics of heterogeneous VCA tissue may be feasible with lower RIN values than currently recommended in standard workflows. The results are summarized in Table 1.

**TABLE 1:** Spatial transcriptomics quality control metrics.

Condition	RIN	Spots under tissue	Median log10(UMIs/spot)	Median log10(genes/spot)	Median log10(genes/UMI)	Median % mito
Condition_1	4.1	3,723	3.764550	3.306639	−0.4475015	12.165725
Condition _2	3.7	4,615	3.691258	3.248464	−0.4372190	7.267751
Condition _3	4.2	3,533	3.846090	3.401573	−0.4453237	4.381405
Condition _4	3.9	3,672	3.765445	3.319418	−0.4437007	3.003134
Condition _5	6.9	3,156	3.312389	3.015988	−0.2873477	4.184338
Condition _6	6.0	4,033	2.949390	2.731589	−0.2089480	5.785513
Condition _7	6.1	2,931	3.518382	3.138934	−0.3745310	3.155790
Condition _8	6.2	3,900	3.239800	2.955206	−0.2781452	3.765504

### 3.2 Cryopreservation is essential for spatial transcriptomics of rat tissue

Comparative analyses of tissue preservation methods revealed markedly superior RNA and cDNA quality in cryopreserved samples compared to formalin-fixed paraffin-embedded (FFPE) specimens. As shown in Figure 3, RNA and cDNA yields were assessed at multiple quality control checkpoints throughout the spatial transcriptomics workflow. During RNA quality control, FFPE samples exhibited strongly reduced ribosomal RNA peaks accompanied by an accumulation of shorter RNA fragments, indicating substantial RNA degradation. In contrast, cryopreserved samples displayed pronounced 18S and 28S ribosomal peaks with overall higher signal intensity, consistent with preserved RNA integrity (Figure 3A). At the cDNA quality control step, cryopreserved samples showed broad cDNA fragment distributions predominantly ranging from 200 to 9,000 bp, consistent with the recommended size range for Visium library preparation. In addition, these samples exhibited a distinct and uniform cDNA peak profile, indicating successful cDNA amplification and cleanup. In contrast, FFPE samples demonstrated fragmented and low-intensity cDNA profiles with minimal representation within the desired fragment range (Figure 3B). Following library construction, cryopreserved samples exhibited a defined library peak at approximately 482 bp, whereas FFPE samples showed little to no detectable library fragments within the expected range of 200–600 bp required for sequencing library generation (Figure 3C).

**FIGURE 3:**
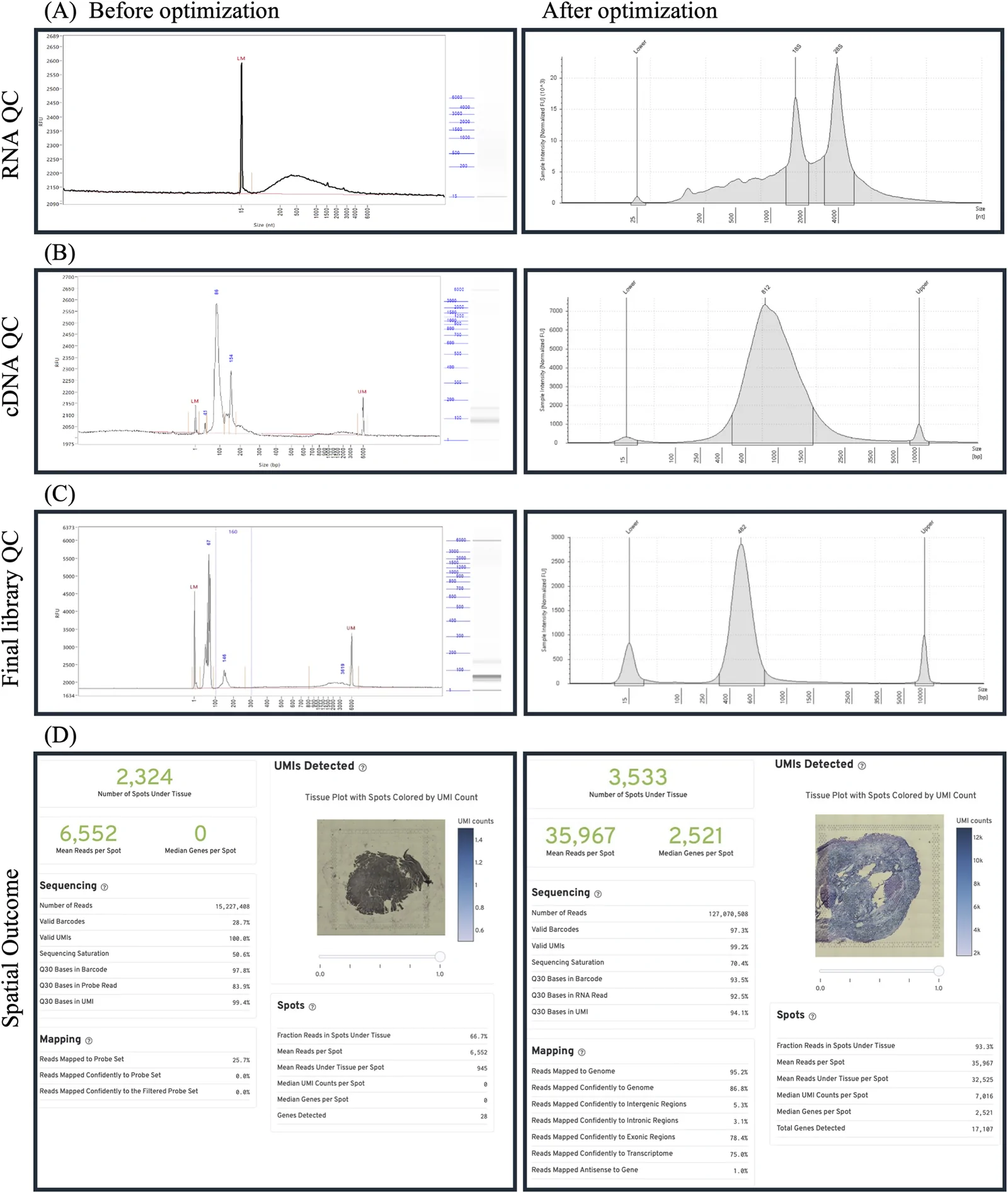
Comparison of RNA, pre-library, post-library, and spatial transcriptomic quality control before (left) and after (right) workflow optimization. Representative Fragment Analyzer and TapeStation electropherograms as well as Space Ranger outputs and quality control metrics show quality profiles at different stages of the workflow. **(A)** RNA integrity assessment comparing FFPE (left) and cryopreserved samples (right). **(B)** Quality control of pre-library cDNA. **(C)** Quality control of final sequencing libraries. **(D)** Representative spatial transcriptomic outputs and quality control metrics from the Space Ranger Web Summary, with representative sample R8 shown on the right.

Overall, FFPE tissue demonstrated insufficient RNA and cDNA preservation for reliable downstream spatial transcriptomics analysis. In contrast, cryopreserved tissue consistently fulfilled quality control requirements throughout the workflow, enabling robust library preparation and high-quality spatial transcriptomics profiling.

### 3.3 Proper freezing workflow is required for section integrity and morphology

As shown in Figure 4, rapid freezing in liquid nitrogen resulted in pronounced freezing artefacts (Figure 4A). In addition, improper handling during cryosectioning led to substantial tissue tearing and structurally inhomogeneous sections (Figure 4B). Preservation of bone within the tissue block further caused severe morphological distortions and mechanical disruption during sectioning (Figure 4C). In contrast, removal of the tibia and fibula by periosteal elevation enabled the generation of uniform, intact cross-sections with well-preserved tissue architecture. Immediate processing after tissue harvest, combined with controlled freezing in an isopropanol bath on dry ice, yielded high-quality H&E images, as illustrated in Figure 4D.

**FIGURE 4:**
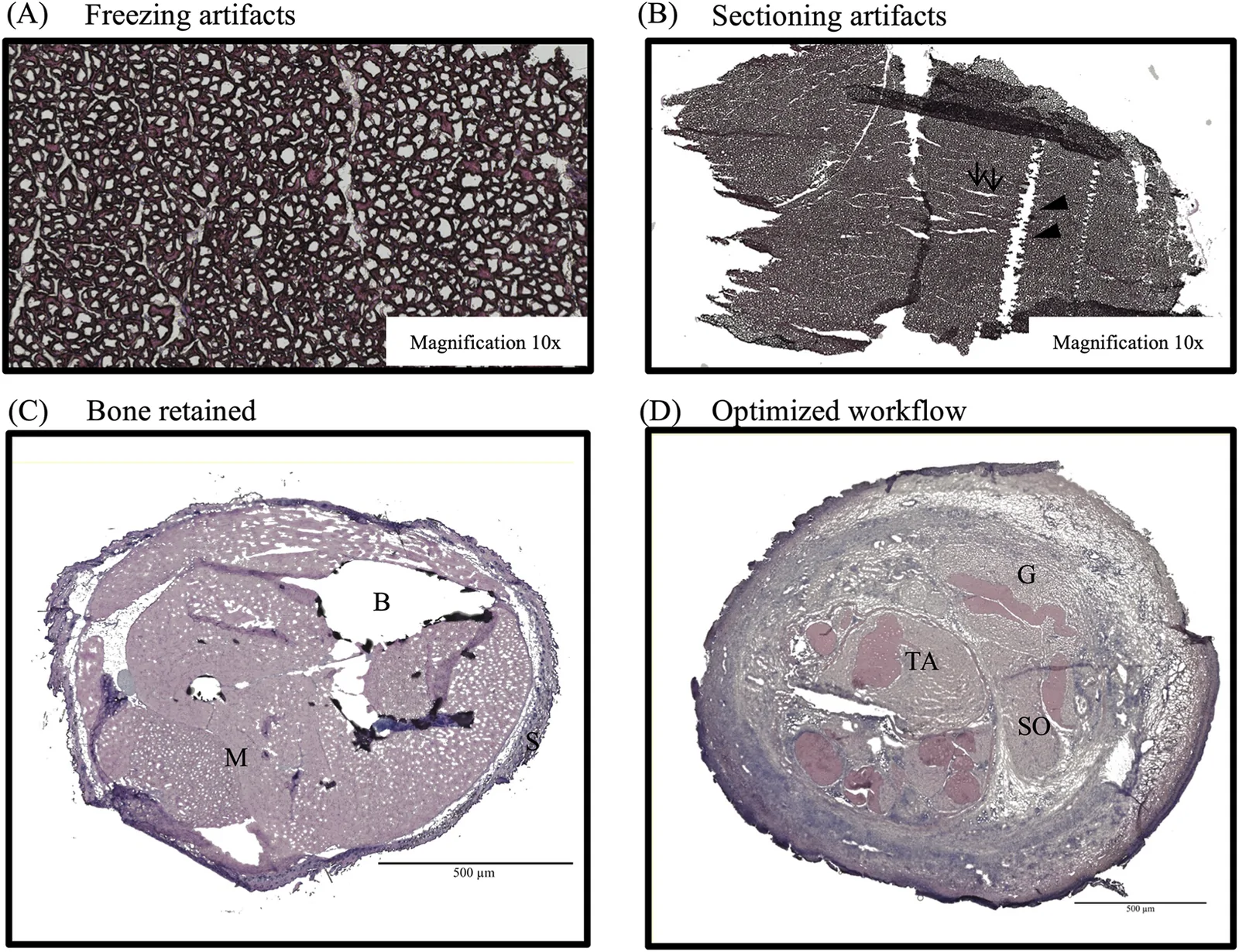
Hematoxylin and eosin (H&E) staining of hindlimb cross-sections illustrating section quality before and after workflow optimization. **(A)** H&E staining of rat tissue demonstrating freezing artifacts caused by rapid freezing and **(B)** sectioning artifacts, including chatter caused by sectioning below the optimal cuttings temperature (arrow) and coarse tissue tears generated during cryosectioning (arrowhead). **(C)** Rat hindlimb tissue section with retained bone, demonstrating impaired section integrity in preliminary experiments. **(D)** Optimized H&E staining of a complete rat hindlimb cross-section after workflow refinement, demonstrating preserved tissue morphology and anatomical orientation; Abbreviations: B, bone-associated sectioning artifact; G, gastrocnemius muscle; M, muscle tissue; S, skin tissue; SO, soleus muscle; TA, tibialis anterior muscle.

### 3.4 Protocol modification enhances immunofluorescence staining of CD68-GFP

Following the modified immunofluorescence protocol, the native GFP signal remained detectable but was weak. Additional staining with an anti-GFP antibody markedly enhanced visualization of GFP-positive recipient-derived cells. Immediate processing of the freshly cut sections was essential to prevent tissue drying. GFP fluorescence was visually compared between samples with and without additional anti-GFP antibody staining. Figures 5A,B demonstrate improved visualization of the GFP signal following antibody-based amplification. Figures 5C,D summarize the optimized immunofluorescence protocol combining 15 min PFA fixation with anti-GFP signal enhancement.

**FIGURE 5:**
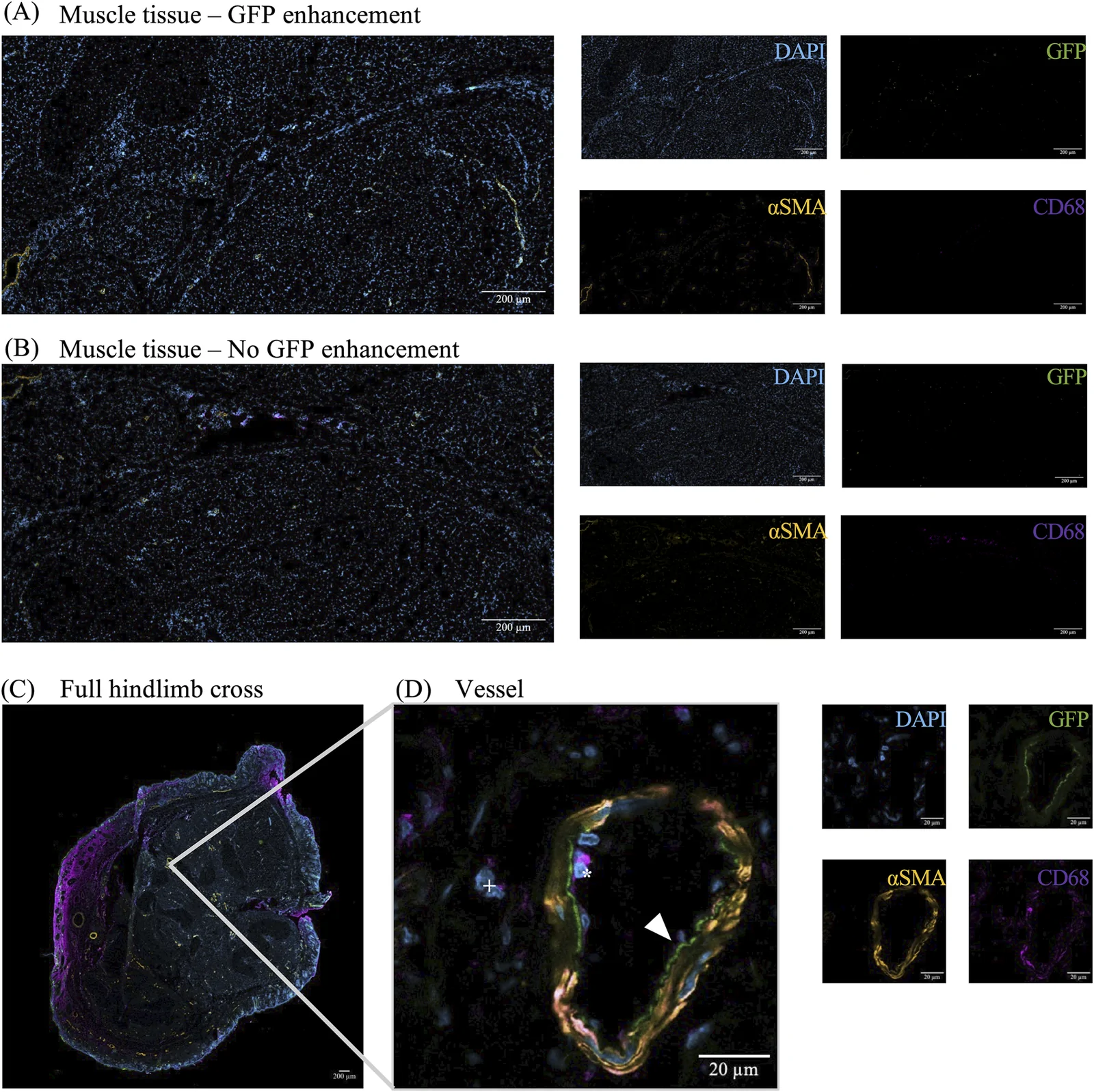
Comparison of immunofluorescence staining of rat hindlimb cross-sections with and without GFP signal enhancement using an anti-GFP antibody. **(A)** Muscle tissue with GFP signal enhancement showing co-staining for αSMA (orange, vessels), anti-GFP (green, recipient-derived monocytes), and ED1 (pink, pan-monocyte marker). The use of the anti-GFP antibody enhanced the visualization of recipient-derived monocytes. **(B)** Muscle tissue without additional GFP signal enhancement, showing co-staining for αSMA (orange, vessels) and ED1 (pink, monocytes). **(C)** Immunofluorescence staining of the entire hindlimb cross-section. **(D)** ×63 magnification of a vessel with recipient-derived monocytes, imaged using LSM980 MP AiryScan 2 confocal microscope. The arrow indicates the vessel boarder, the asterisk (*) indicates an intravascular monocyte, and the plus sign (+) indicates a tissue-infiltrating monocyte.

### 3.5 Tissue permeabilization time was optimized for heterogeneous hindlimb cross sections

Tissue permeabilization required careful optimization due to the heterogeneous composition of the hindlimb cross sections. As permeabilization efficiency is tissue-dependent, accurate incubation timing was essential to balance efficient RNA release with preservation of spatial signal integrity. The optimal permeabilization time was determined by comparing the fluorescence intensity of cDNA capture spots with that of the corresponding H&E-stained sections. Permeabilization times of 30, 33, 36, 42, and 48 min were tested for rat hindlimb tissue. As shown in Figure 2, the sample permeabilized for 33 min exhibited the highest fluorescence intensity and the lowest signal diffusion, indicating optimal RNA release for downstream spatial transcriptomics analysis. Semiquantitative image analysis in ImageJ supported the visual assessment, with the 33 min condition showing the highest percentage of fluorescence-positive area (32.4%) compared with the 36 (9.2%) and 42 min (17.4%) conditions.

### 3.6 All samples met spatial transcriptomics quality control criteria

Spatial transcriptomic quality control metrics were within the expected range across all samples. Median log_10_ UMIs per spot ranged from 2.95 to 3.85 and log_10_ genes per spot ranged from 2.73 to 3.40. The genes-per-UMI ratio indicated adequate transcript complexity and mitochondrial read percentage was generally low (3.00%–12.17%), consistent with acceptable RNA integrity and tissue preservation. Although variability between individual samples was observed, all datasets showed robust quality metrics compatible with downstream spatial transcriptomic analysis. The workflow was robust across different experimental conditions, including control, acute and chronic rejection settings (Table 1; Figures 6A–D). Based on the optimization steps, the resulting recommended workflow and critical procedural considerations are summarized in Figure 6E.

**FIGURE 6:**
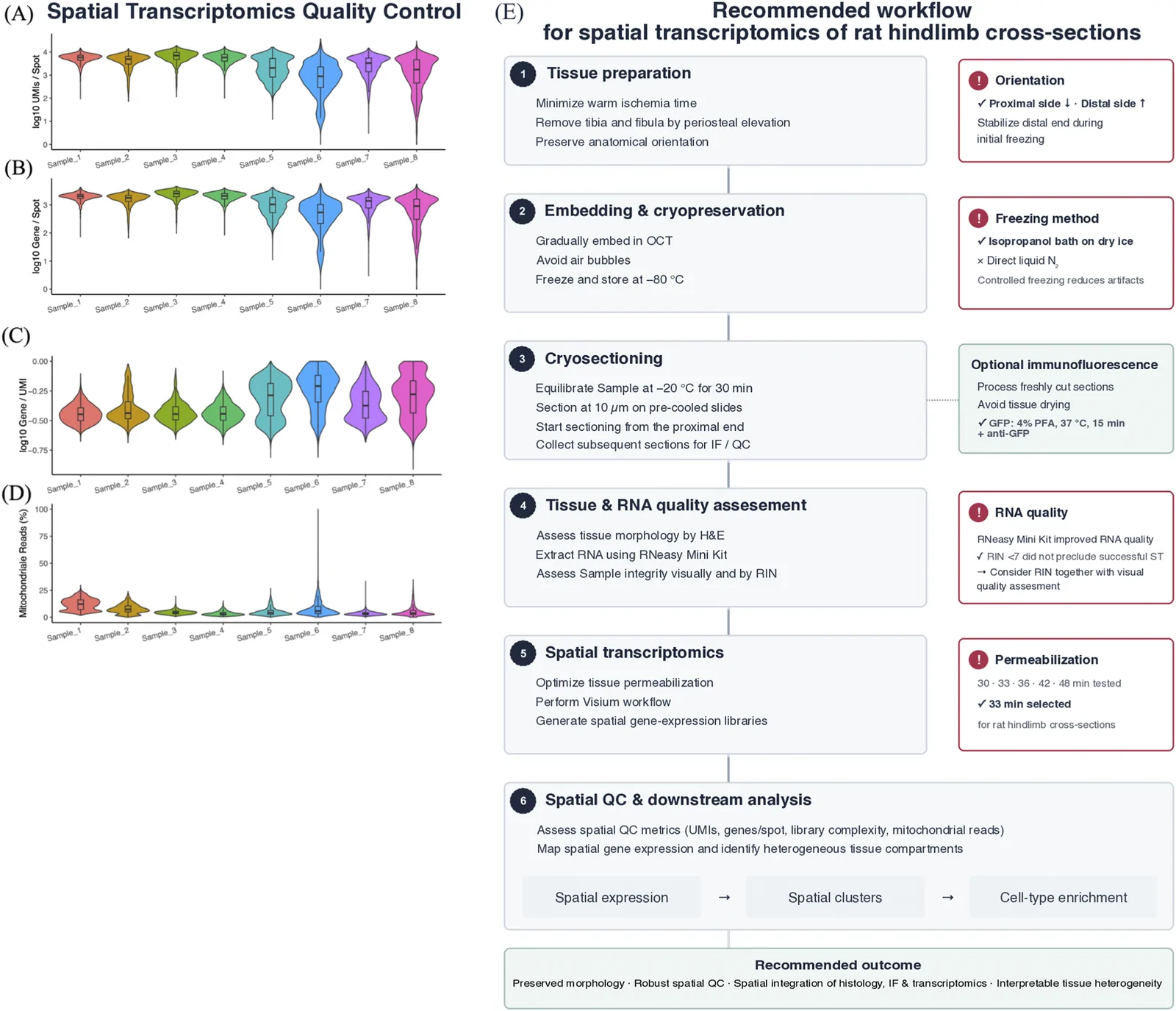
Spatial transcriptomics quality control. Violin plots summarize **(A)** the transcript counts (log_10_ UMIs per spot), **(B)** the gene counts (log_10_ genes per spot), **(C)** the library complexity (log_10_ genes per UMI) and **(D)** the percentage of mitochondrial reads across all analyzed conditions. **(E)** Summary of the optimized workflow and critical decision points for spatial transcriptomics of rat hindlimb cross-sections. The workflow highlights tissue preparation and orientation, embedding and controlled cryopreservation, cryosectioning, tissue and RNA quality assessment, tissue-specific permeabilization, spatial quality control and downstream analysis. Optional immunofluorescence of adjacent sections enables integration of histological, immunofluorescence and spatial transcriptomic information.

### 3.7 Spatial transcriptomics resolves heterogeneous tissue compartments

Downstream spatial transcriptomics analysis was performed across all samples to assess spatial gene expression and transcriptional heterogeneity. To illustrate the different data layers and downstream analyses enabled by the optimized workflow, sample R8 is shown as a representative example in Figure 7.

**FIGURE 7:**
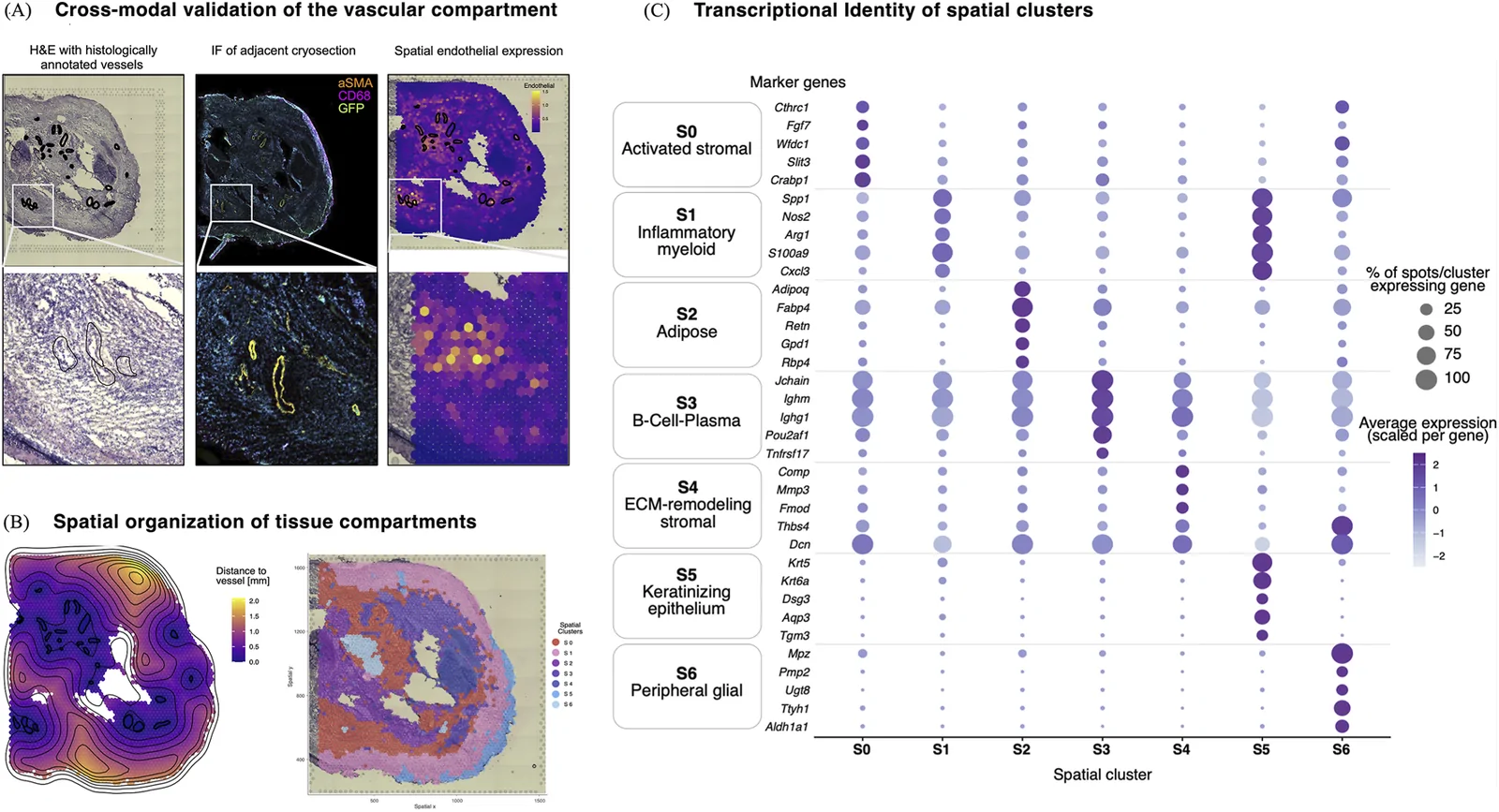
Spatial characterization of heterogeneous tissue compartments. **(A)** Cross-modal comparison of the vascular compartment in representative sample R8. Histologically annotated vascular structures in H&E-stained tissue are compared with α-SMA immunofluorescence in an adjacent cryosection and spatial endothelial enrichment derived from single-cell reference-based integration. Lower panels show enlarged views of the corresponding regions of interest. **(B)** Spatial organization of tissue compartments. The distance to histologically annotated vessels (left) and unsupervised spatial clustering identifying seven transcriptional clusters (S0–S6) (right) are shown. **(C)** Transcriptional identity of the spatial clusters. Five representative marker genes are shown for each cluster. Dot size indicates the percentage of spots expressing the respective gene, while color represents average expression scaled across clusters for each gene. Marker gene profiles are consistent with activated stromal (S0), inflammatory myeloid (S1), adipose (S2), B-cell/plasma (S3), ECM-remodeling stromal (S4), keratinizing epithelial (S5), and peripheral glial (S6) tissue compartments.

Histologically annotated vascular structures were compared with α-SMA immunofluorescence in an adjacent cryosection and spatial endothelial enrichment. As shown in Figure 7A, these complementary data layers demonstrated spatial correspondence of the vascular compartment. The spatial relationship to histologically annotated vessels was additionally visualized using a vessel-distance map (Figure 7B).

Unsupervised clustering of the spatial transcriptomics data identified seven spatially organized transcriptional clusters (S0–S6) in the representative sample R8 (Figure 7B). Differential expression analysis revealed distinct marker gene profiles across these clusters (Figure 7C), consistent with activated stromal (S0), inflammatory myeloid (S1), adipose (S2), B-cell/plasma (S3), ECM-remodeling stromal (S4), keratinizing epithelial (S5), and peripheral glial (S6) tissue compartments.

Together, these findings demonstrate that the optimized workflow generates spatial transcriptomics data that retain biologically interpretable spatial and transcriptional heterogeneity and allow the distinction of heterogeneous tissue compartments beyond standard quality control metrics.

## 4 Discussion

This study presents an optimized workflow enabling high-quality spatial transcriptomics and immunofluorescence analysis in large, heterogeneous VCA tissue of rat hindlimb. By systematically adapting critical steps, including cryopreservation, bone removal, permeabilization timing, RNA extraction, and GFP signal preservation, we addressed several methodological limitations that previously prevented reliable spatial analysis of whole-limb allografts. Furthermore, by preserving endogenous GFP signals and improving RNA quality, this protocol allows the integration of spatial transcriptomics with reporter-based immunofluorescence, enabling direct visualization and transcriptional characterization of recipient-derived monocytes and macrophages within the allograft.

Initial experiments using FFPE-preserved tissue yielded highly degraded RNA and insufficient transcriptomic signal, consistent with known RNA fragmentation in formalin-fixed samples (Srinivasan et al., 2002). Transition to a fresh-frozen embedding protocol markedly improved RNA preservation and data quality. Cryopreservation maintains the integrity of poly(A) tails required for capture by spatial barcoded oligonucleotides and proved essential for successful spatial transcriptomic analysis in this large composite tissue. Although the Visium spatial transcriptomics kits were originally developed for mouse tissue, they have been successfully applied to rat samples, including rat heart tissue, supporting their suitability for the use in rat hindlimb VCA model (Mesfin et al., 2025).

RNA integrity, measured by the RNA Integrity Number (RIN), is a key parameter for successful spatial transcriptomics performance. RNA quality depends strongly on sample handling and embedding conditions (Liu et al., 2022). According to 10x Genomics, a RIN ≥ 7 is recommended for high-quality spatial transcriptomic data (10x Genomics, 2023). However, preservation of RNA integrity must be balanced against slower, controlled freezing conditions required to minimize morphological artifacts in complex tissue. Furthermore, RNA integrity obtained during later extraction depends on both the chosen isolation method and meticulous handling; inappropriate processing can lead to artificially low RIN values that do not reflect true RNA quality. This consideration may be particularly relevant for heterogeneous and structurally complex tissue such as VCA samples. In the present study, Qiagen-based RNA isolation achieved RIN values of up to 6.9, slightly below the recommended threshold, yet downstream quality control demonstrated robust cDNA quality and successful spatial transcriptomic processing. These findings suggest that conventional RIN cutoffs may be overly restrictive for heterogeneous VCA tissue and that transcriptomic suitability cannot be assessed solely based on extracted RNA integrity measurements.

Cryosectioning posed additional challenges due to the heterogeneous mechanical properties of muscle, skin, connective tissue, and dense bone. Bone retention consistently resulted in shearing and incomplete sections. Removal of the tibia and fibula prior to embedding was therefore essential to achieve coherent, anatomically preserved sections suitable for histological and transcriptomic processing. Another unique feature of this study was the use of hCD68-GFP transgenic recipient rats, enabling spatial visualization of monocytes. A 15-min fixation in pre-warmed 4% PFA combined with antibody-based signal amplification enabled reliable visualization of recipient-derived monocytes. Optimizing the permeabilization step to 33 min ensured sufficient RNA release across all tissue compartments while preventing overpermeabilization and RNA diffusion. Spatial transcriptomics quality control metrics were within the expected range for all samples, although variability between experimental groups was observed. Notably, samples with lower RIN values still demonstrated adequate spatial quality control performance, indicating that RIN alone may not fully predict suitability for spatial transcriptomic analysis. This observation supports the notion that RNA extraction methodology can influence RIN values without necessarily reflecting true RNA degradation within the tissue. Reduced transcript diversity may reflect differences in tissue composition rather than technical limitations of the workflow. Differences in library complexity and mitochondrial read percentages were detected between the samples, however all datasets remained within acceptable thresholds for reliable downstream analysis. The workflow therefore proved robust across diverse tissue conditions and experimental settings.

Several trade-offs must be acknowledged. Bone removal prior to freezing significantly improved section quality and morphology but excluded the osseous component for downstream spatial transcriptomic analysis. Consequently, no RNA or spatial gene expression data could be obtained from bone tissue, and minor alterations in cross-sectional anatomy were observed due to traction of surrounding tissue. Furthermore, freezing in isopropanol on dry ice (−78.4 °C) was slower than freezing in liquid nitrogen (−196 °C), which may have slightly increased the risk of RNA degradation. However, this approach yielded more uniform tissue preservation, minimized freezing artifacts, and provided better adhesion on slides and improved histological image quality. Consecutive cryosections were used for spatial transcriptomics and immunofluorescence analysis. Although the sections were only 10 µm apart, minor structural differences or handling artifacts can limit direct co-localization of transcripts and protein signals. Therefore, the immunofluorescence section should be regarded as a complementary validation tool for spatial localization of cell markers. Finally, as spatial transcriptomics provides near-cellular but not single-cell resolution and immunofluorescence staining and spatial transcriptomics were performed on consecutive rather than identical sections, precise one-to-one transcript-to-protein colocalization was not possible.

Importantly, the principles established here are not restricted to rat hindlimb VCAs. The optimized workflow for controlled sample preparation, gradual freezing, optimized permeabilization and careful fixation can be readily transferred to other large and complex tissues with or without endogenous fluorescence signals, such as musculoskeletal samples, peripheral nerve, or organ transplant specimens.

While single-cell and single-nucleus transcriptomic approaches have been applied to individual rat tissue compartments, including skeletal muscle, to our knowledge they have not yet been reported specifically for rat hindlimb transplantation (Hettinger et al., 2022). Tissue dissociation in conventional single-cell approaches results in loss of the native spatial relationships between different tissue compartments. In contrast, the spatial transcriptomic approach presented here preserves multiple tissue types within the same hindlimb cross-section, enabling their transcriptional characterization while retaining their anatomical context. Existing single-cell datasets nevertheless provide valuable reference information for cellular annotation; accordingly, the published *R. norvegicus* single-cell transcriptomic dataset by Ma et al. (2020) was used as a reference for cell-type integration in the present study.

Furthermore, this protocol establishes a robust methodological foundation for future studies investigating acute and chronic rejection in vascularized composite allotransplantation. Several key methodological adaptations were identified as critical for successful spatial transcriptomic analysis of heterogeneous VCA tissue. Controlled slow freezing in an isopropanol bath was essential for preserving tissue morphology, while removal of the tibia and fibula substantially improved cryosectioning quality and tissue integrity. In addition, the optimized permeabilization time of 33 min enabled efficient RNA release while minimizing signal diffusion. For reliable visualization of GFP-positive monocytes, additional anti-GFP immunofluorescence staining proved necessary to enhance endogenous GFP signal detection. Our findings further suggest that comparatively low RIN values do not necessarily preclude successful downstream spatial transcriptomic analysis in complex VCA tissue. The present workflow can be applied to explore immune cell infiltration, macrophage polarization, and molecular pathways underlying chronic rejection at the transcriptional level. The combination of spatial transcriptomics and immunofluorescence provides an integrative approach for studying immune dynamics and tissue remodeling in complex transplant environments.

In conclusion, we present the first workflow enabling high-quality spatial transcriptomic and immunofluorescence analyses of whole-limb rat VCA cross sections. It provides a reproducible and transferable framework for future studies of complex transplant tissue and may facilitate improved understanding of acute and chronic rejection processes in vascularized composite allotransplantation.

## Data Availability

The original spatial transcriptomics data for the representative sample R8 are publicly available in Figshare at doi:10.6084/m9.figshare.34557231.
